# A QoS Optimization Approach in Cognitive Body Area Networks for Healthcare Applications

**DOI:** 10.3390/s17040780

**Published:** 2017-04-06

**Authors:** Tauseef Ahmed, Yannick Le Moullec

**Affiliations:** Thomas Johann Seebeck Department of Electronics, Tallinn University of Technology, Ehitajate tee 5, 19086 Tallinn, Estonia; yannick.lemoullec@ttu.ee

**Keywords:** cognitive body area network, reinforcement learning, channel allocation, power allocation, channel model, wireless body area network

## Abstract

Wireless body area networks are increasingly featuring cognitive capabilities. This work deals with the emerging concept of cognitive body area networks. In particular, the paper addresses two important issues, namely spectrum sharing and interferences. We propose methods for channel and power allocation. The former builds upon a reinforcement learning mechanism, whereas the latter is based on convex optimization. Furthermore, we also propose a mathematical channel model for off-body communication links in line with the IEEE 802.15.6 standard. Simulation results for a nursing home scenario show that the proposed approach yields the best performance in terms of throughput and QoS for dynamic environments. For example, in a highly demanding scenario our approach can provide throughput up to 7 Mbps, while giving an average of 97.2% of time QoS satisfaction in terms of throughput. Simulation results also show that the power optimization algorithm enables reducing transmission power by approximately 4.5 dBm, thereby sensibly and significantly reducing interference.

## 1. Introduction

Recent advances in electronics and system on chip design have given birth to a new era of wireless communication systems. Intelligent and low powered devices enable building small and miniaturized wireless networks to help and improve human life, including among other things, wireless body area networks (WBANs). A WBAN is a combination of ultra-low powered, programmable miniaturized sensor nodes combined with wireless radio communication capabilities used to monitor the human body and its physiological features, e.g., heart rate, blood pressure, temperature, etc., using either invasive, or preferably, non-invasive sensors [1,2,3].

WBANs are touted as one of the key technologies foreseen to help dealing with the challenges found in many healthcare systems around the world. Indeed, current healthcare facilities are facing huge challenges from the growing elderly population and limited human and financial resources. In the USA alone, from 1960 to 2010, life expectancy increased on average by 13.5% [4]. The population aged 60–80 years will have doubled by 2050 (from 33 million to 81 million people) [5]. It is expected that this huge increase will overload the healthcare system and overall quality of life will be affected. Let us consider a patient who has to visit a doctor for a routine blood pressure and temperature check; for this, he/she has to travel to the health facilities. This is costly in terms of time and money and overloads the healthcare system; there are more urgent health issues and cases that must be taken care of by health practitioners. To avoid such situations, a remote system can enable the persons to send information about their health status to health care givers from their homes and thus they do not need to get out from their comfort zone just to do routine monitoring. This will both greatly benefit the overall health care system and quality of life in general.

A WBAN allows the integration of intelligent small sensor nodes in, on, or around the human body to monitor the human vital signs. The miniature devices are basically small radios that can collect data through their sensors and transmit them to a central node or a master node. This central node can be linked to a backend network where, upon reception, the patients’ data can be analyzed for diagnosis and prescriptions. Generally, a WBAN can consist of in-body and/or on-body area networks. An in-body area network allows communication between invasive or implanted sensor nodes to a base station. An on-body area network, on the other hand, allows communication between a non-invasive or wearable sensory device to a base station. Since human tissues are being monitored, the devices used in WBAN have to build upon ultra-low power radio transmitters so as to avoid any adverse effects on the human health.

In this paper, we focus solely on on-body area networks where single or multiple sensors are attached or fitted to a person. A key idea is that instead of having all the on-body sensors transmitting to the base station individually, a central on-body node is used as an intermediate hub and receives all the sensors’ data and transmit them to the base station. From here onwards, we will refer to such a sensing device as the node which is routing all the sensors’ data to the base station. The base station itself will be referred to as gateway from here onwards.

Although wireless sensor networks in general are not new per se, their design and capabilities are continuously evolving, including the adoption (and adaptation) of certain cognitive elements previously developed for cognitive radios [6,7] either for resource allocation (frequency, power, etc.) and/or signal processing and data analytics. Cognitive capabilities can be exploited in smart routing and provide the ability to foresee any changes in the routing path [8]. Such a trend is also being applied to WBANs and result in what we term cognitive body area network (C-BAN). In this work, we address one of the major issues in such a setup, i.e., the tradeoff between throughput and quality of service (QoS) by means of a cognitive and dynamic approach for frequency allocation. This approach, based on re-enforcement learning, is named RL-CAA (for Reinforcement Learning—Channel Assignment Algorithm). The specific contributions presented in this paper are:

- A context aware channel allocation RL-CAA approach in which the radio environment is first sensed, and based on the feedback received in the form of signal to noise ratio (SNR), the approach decides the fate of the channel. If the channel has a better SNR than other available channels, and if there is no co-channel interference, the channel is assigned to that particular gateway. Based on the throughput requirement, one or more channels can be assigned. The algorithm is robust to traffic changes; QoS satisfaction is its main objective.
- The IEEE 802.15.6 standard has not formulated an off-body mathematical channel model. However, the IEEE standard document provides experimented values that can be used as the basis for such a model. Based on those experimental results, we have formulated a mathematical model for the off-body Channel Model 4 (CM4) using MATLAB that takes on-body posture and shadowing effects into account.
- We propose a novel power allocation algorithm that aims at achieving the desired interference level at the boundary of the gateway’s coverage area. Its primary convergence criterion is interference minimization towards other gateways.

The rest of this paper is organized as follows: the current state of the art related to WBANs is described in Section 2. In Section 3, the detailed system model is presented and our simulation scenario is described. The simulation results are presented and discussed in Section 4. Finally, the last section concludes the paper.

## 2. Related Works

WBANs have become a popular research topic in the last few years. Despite WBANs’ potential uses in many fields of human life, they are not yet widely adopted and further research and development are still needed. There are many open research questions and challenges that remain to be addressed. The authors of [9] have presented a comprehensive overview of WBANs. They discussed the network architecture, sensor hardware, 802.15.6 IEEE standard layers [10] and emerging radio technologies available for WBAN applications. They also presented a taxonomy of proposed WBAN projects. They highlighted various open research challenges related to the bandwidth and energy efficient protocols, issues arising from the co-existence of WBANs with other wireless technologies and WBANs’ successful integration into society. The authors in [11] have presented an overview of WBAN applications related to healthcare. They recommended various wireless technologies for WBAN medical applications; however, the non-medical aspects of WBANs were not considered and no specific details for designing a WBAN application were presented. In [12], the authors described the prospective usage of WBANs in the future health care system. In [2,12,13], the authors have provided extensive overviews of generic WBANs and their applications, but they fall short in addressing specific research challenges. Like with any other wireless network, interference is a major hindrance in WBANs. Interference occurs commonly if multiple WBANs are deployed next to each other in a given physical location. Inter-network interference can seriously diminish the performance of WBANs if proper interference avoidance methods are not considered. In [14], the authors presented an approach towards inter-network inference mitigation while considering the energy efficiency of WBANs. A power control algorithm was presented for interference mitigation and avoidance. The authors presented simulation results to prove their hypothesis. WBANs are like cognitive radios in the sense that they share the spectrum with other existing technologies such as Bluetooth, ZigBee, Wi-Fi, etc., [12]. In [14], the authors did not mention any interactions, either positive or negative, with other technologies which share the spectrum bands with WBANs. In [15], the authors described the convergence of cognitive radios (CR) and internet of things (IoT), i.e., an overview of how CR technology can be beneficial for various sensor network fields. There may be possibilities for WBANs to also interact with the internet directly at some tier of the communication stack. However, that paper did not present any specific system design or implementation suggestions. In addition to the internet, WBANs may also interact with other existing wireless communication technologies and standards. In [16,17], the authors described the integration of health care system WBANs with other communication technology standards and protocols and they reviewed their aspects. The authors in [16] proposed a unified networking model for hospital scenarios combing various networking technologies. They have analyzed the basic requirements for such systems. Each networking system relies on a wired backbone network and a detailed analysis highlights how combining wired and wireless network would really give the future hospital network designers a clear picture. The authors in [17] proposed a hybrid model of WBAN data transmission and sharing. Their proposed architecture combines WBAN communication tiers with the cloud for data sharing and delivery in healthcare applications. However, including the cloud may pose some data security challenges that are now being acknowledged by this research community as a critical issue.

Since WBANs operate in a traffic changing and interfering environment, many distributed WBANs can be located in the same vicinity and there could be other communication technologies (e.g., Bluetooth, ZigBee, etc.) communicating in the same spectrum bands. This could give rise to a competition for the available spectrum, i.e., channels. Hence, there must be mechanisms for channel allocation and sharing in such a way to satisfy the traffic and throughput requirements. Another constraint for operating in such a heterogeneous environment is interference. To avoid interference, some power control mechanism must be incorporated in the WBANs. In [18], the authors have presented a channel sharing protocol for WBANs, but only for medical applications. Therefore, such an approach lacks information about spectrum sharing among networks to facilitate their co-existence in the available spectrum. In [19], the authors investigated channel allocation schemes in general for a wireless sensor network (WSN) based on game theory. The authors considered a general WSN for investigating the behavior of their algorithm, showing that it exploits the network knowledge to reduce interference and improve overall WSN performance. Such an approach could lead to additional studies for WBANs. The authors in [20] proposed a multichannel MAC coordination framework called decentralized time synchronized channel swapping. The proposed protocol combines the benefits of decentralized time division multiple access and spontaneous alignment of nodes’ time slots across channels and flexible time synchronization by instantaneous adoption of available slots. The authors compared their proposed MAC protocol for channel swapping with time synchronous channel hopping and efficient multichannel MAC protocols. Simulation and experimental results show that the proposed approach leads to a significant reduction in convergence time and provides higher network throughput with and without the presence of interference. However, the proposed scheme has been applied to ad hoc wireless networks and the behavior of the protocol in WSNs and WBANs is yet to be investigated.

In [21], the authors describe channel estimation and power control schemes for WBANs. The authors claim that their proposed algorithm can save up to 25% of energy as compared to a fixed transmission power scheme. The proposed algorithm has been investigated in IEEE 802.15.6 beacon mode [22] with a single hop star topology. In [23], the author investigated transmission power assignment schemes and energy minimization techniques for WBANs. The author used two strategies to assign transmission powers to the on-body sensor nodes; with or without body posture state information. He studied his power optimization approach in a single hop transmission link. However, the proposed transmission power assignment algorithms presented in [21,23] have not been investigated nor evaluated for the specific off-body communication links used in WBANs and their potential impact in a two-hop star topology.

The person or patient who is being monitored cannot always remain stationary, i.e., laying in bed or sitting. Hence, there is a need for mobility algorithms to be implemented into the WBAN so that seamless communication occurs when the nodes are mobile and they move from one access point to another access point. In [24], the authors presented a seamless mobility and handover scheme for WSNs taking into account the sensor mobility from one access point to another. This approach is used as the basis for our own handover implementation described in Section 3.4. The authors in [25] have presented an optimization algorithm for the design of WBANs. The approach is based on a heuristic model and an ant colony optimization method. The authors proposed these algorithms for WBAN design considerations, i.e., network topology and routing, taking into account the variable data rates from individual sensor nodes. The experimental results are promising in terms of quality and computational time as compared to a commercial optimization problem solver. However, the algorithm needs further investigation to improve its performance.

In [26] a robust optimization approach to tackle traffic uncertainty in network design problems was proposed. The described algorithm is a hybrid heuristic approach based on ant colony optimization. The authors compared the proposed algorithm with a commercial optimization problem solver and the experimental results show that their algorithm gave high quality solutions with low optimality gaps. The authors in [26] considered a general network although the impact of their hybrid optimization algorithm in WBAN design applications needs further research. The authors in [27] proposed a global routing protocol for WBANs which targets optimizing the energy consumption at the nodes and increasing the network lifetime. Experimental and simulation results show a significant increase in network lifetime by balancing the energy consumption across all the network. This is quite advantageous in the sense that all nodes can be replaced or recharged at the same time. However, the proposed protocol only takes into account the transmission power for energy optimization. The protocol needs further investigation to take into consideration more parameters in order to better conserve energy.

## 3. System Model

### 3.1. WBAN Architecture and Requirements

**Figure 1:**
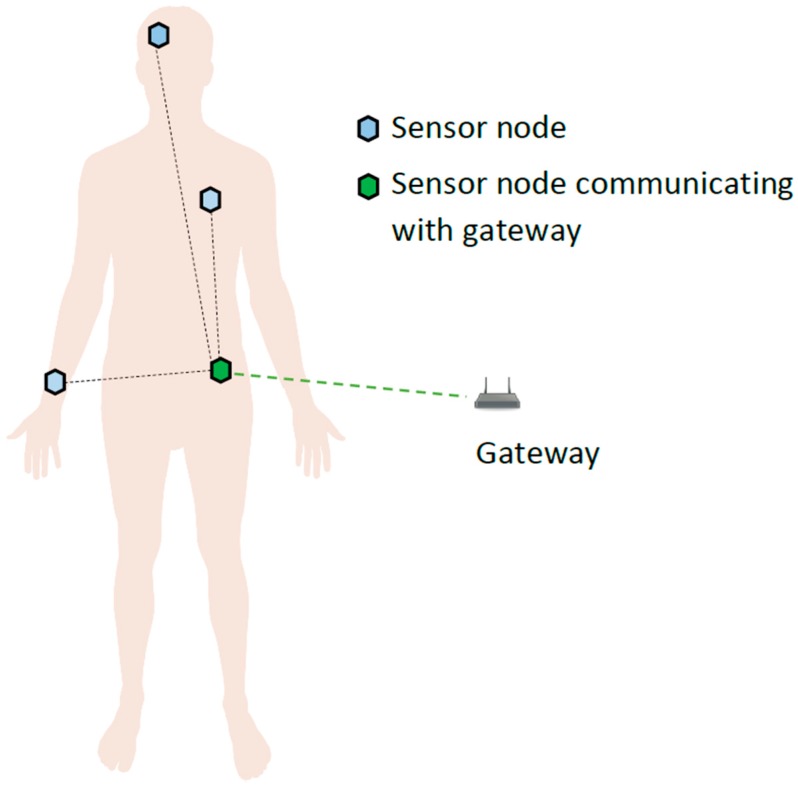
Wireless body area network.

In this section, we present the WBAN model and the communication tier that has been used in this work. A nursing home has been considered as the application scenario. The real dimensions of the physical space can vary, but for simplicity, we consider a hall 10 m in width and 10 m in length. This area has been logically partitioned into smaller sections, called ‘zones’. Each zone has its own gateway acting as a base station, giving wireless coverage in a 5 × 5 m^2^ area. The gateway is fixed (stationary) at the center of the zone and it is assumed that it has a fixed source of power, i.e., wall power. The nodes in each zone correspond to individual patients. A patient can have multiple sensors fitted or attached to his/her body. All these sensors are sending their data to the central sensor node located at waist height; this central node periodically transmits the collected data to the gateway. This concept is shown in Figure 1.

**Figure 2:**
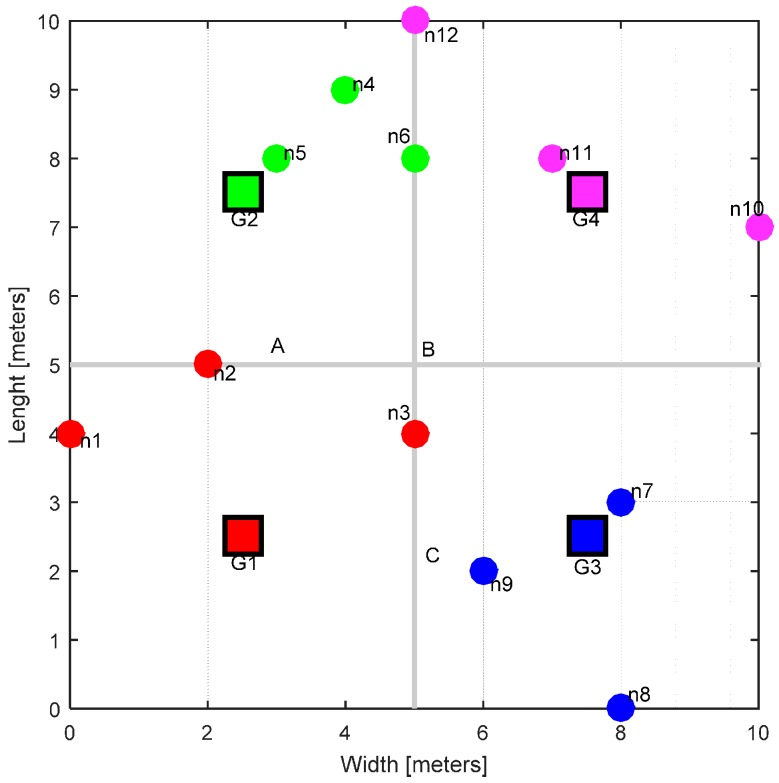
Physical layout and WBANs deployment. The colors show the associations between the nodes and gateways.

The overall hall, zones, gateways and the corresponding nodes are illustrated in Figure 2 (here at the start of the simulation, right after they have been deployed). In this work, we consider the IEEE 802.15.6 standard that specifically targets WBANs [22]. To accommodate the huge application range of WBANs, this standard defines three physical layer options for WBANs, namely narrowband (NB), ultra-wide band (UWB) and human body communications (HBC) [22]. Each physical layer standard has its own design requirements. In this work, we consider only the NB physical layer on the 2.4–2.483 GHz band. This band is termed the industrial and medical (ISM) band. Each gateway is monitoring this band in the 2.4–2.483 GHz range. Other requirements and features of the system are given in Table 1 [22,28].

For simplicity, we use only 20 channels in our simulations. The range of possible data rates for IEEE 802.15.6-compliant WBANs is quite wide (121.4 Kbps–971 Kbps), but to evaluate the system under demanding requirement conditions, we have chosen a data rate of 971.4 kbps in our simulations.

### 3.2. WBAN Network Topology

**Figure 3:**
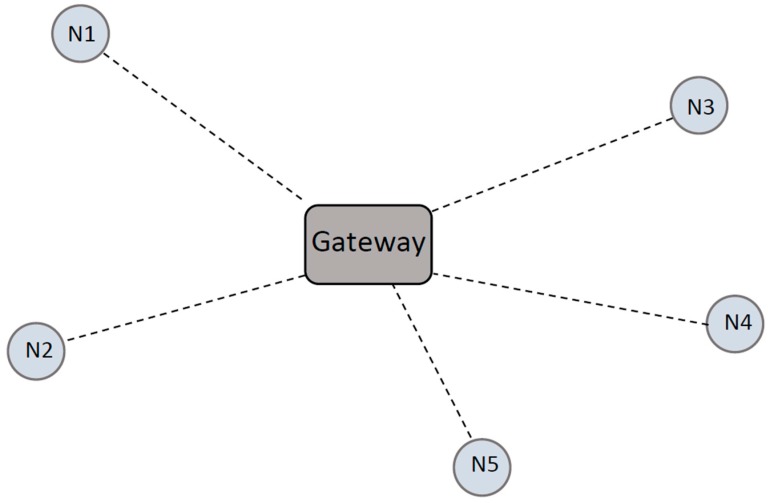
Network topology.

IEEE 802.15.6 considers WBANs to operate in either a one-hop or a two-hop star topology, with the central node to be located at the center of the body, e.g., at waist level, as shown in Figure 1 [22]. Two types of transmissions exist in the one-hop star topology: (A) transmissions from the sensor device to the gateway and (B) from the gateway to the sensor device. In the two-hop star topology, the sensor nodes connect to the gateway via their peer devices or multi-hopping is used to send data to the gateway [29]. In our system model, the two-hop star topology exists between sensor nodes carried by the patient and the gateway via the central node at the waist (Figure 1). We assume that the data sent by the central node is the collection of data from the other sensors. However, each central node carried by an individual person is connected to the gateway via the one-hop star topology, as shown in Figure 3, where N corresponds to the nodes and they communicate with the gateway. Since our research focuses on the spectrum optimization, the analysis of the individual sensor’s data (two-hop star topology mentioned above) is not in the scope of this paper.

The time division multiple access (TDMA) technique is used for medium access. Each node is assigned one time slot; hence, all nodes’ data are combined into a superframe for transmission from the gateway to the backend network for further data processing and/or diagnosis purposes.

### 3.3. Channel Model

**Figure 4:**
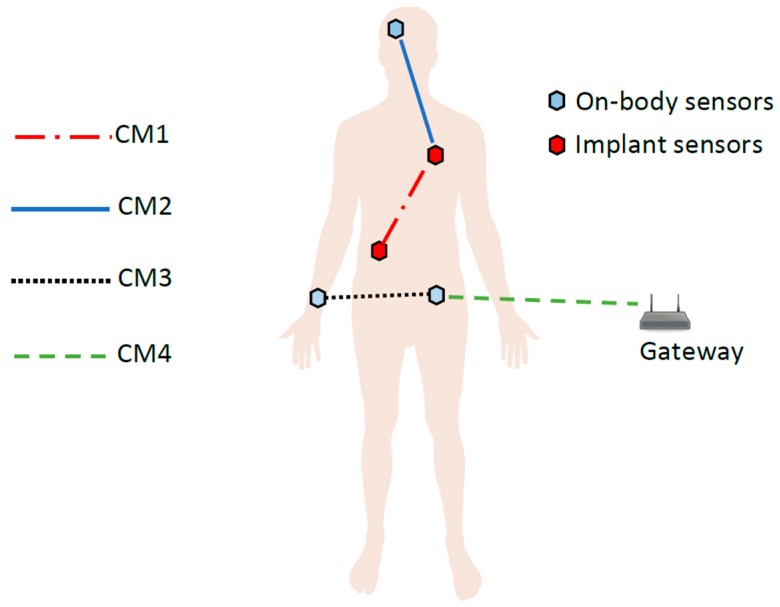
IEEE 802.15.6 Channel Models.

The IEEE 802.15.6 standard document defines the channel models for the WBANs [10]. Figure 4 shows these channels (termed CM1–CM4). As mentioned previously, this paper only addresses the off-body communication, i.e., between the central node (on waist, see Figure 1) and the gateway (Figure 3). This channel is referred to as Channel Model 4 (CM4) in the IEEE standard document [10]. In WBANs, there are many factors which affect and deteriorate the signal quality, e.g., shadowing, reflections, diffractions, interferences, etc. Shadowing is a key factor which causes signal degradation due to the body environment; body movements and postures can also cause shadowing. Therefore, the node is either in direct line of sight (LOS) with the gateway or in non-line of sight (NLOS) at any instant. This also creates additional complications to create an accurate mathematical model for CM4. The IEEE standardization document presents the measured values for the channel model [10] but no concrete mathematical model is included. From these measured values, we have formulated a mathematical channel model for the CM 4 using MATLAB, as shown in Equation (1):

(1) $$\mathrm{pathloss}={\mathrm{ax}}^{3}+{\mathrm{bx}}^{2}+\mathrm{cx}+d,$$

which is a third degree polynomial equation modelling CM4. The identified values for the constants a, b, c and d are given in Table 2.

Our mathematical model for CM4 takes the shadowing caused by body orientation as well as LOS and NLOS into consideration. This mathematical model gives realistic channel properties for CM4 and is in compliance with the values presented in [10].

### 3.4. Mobility and Handovers

The permissible radio range of CM4 is ~5 m (as indicated in the IEEE standard itself [10]). This is the motivation for dividing the coverage area into smaller partitions, i.e., zones of 5 × 5 m^2^. This inspiration of dividing the physical location into smaller regions comes from the cellular networks (e.g., micro or femto cells).

The positions of the gateways are fixed, i.e., at the center of each zone, while the nodes are randomly deployed. All nodes (patients) can freely and randomly move in all directions across all zones, at a constant speed of 1 m per second. The gateways are installed in such a way that when a node crosses the coverage of one gateway, it enters into the other gateway coverage zone, and handover is taking place. A simple distance-based handover algorithm has been implemented to facilitate the movement of all nodes freely across all zones and providing seamless vital data transfer while moving from one access point (gateway) to another [24,30].

### 3.5. Channel Allocations

The gateway is the master node or base station which undertakes the spectrum-related tasks along with data processing and routing. The nodes are sensors which sense and transmit data. Each gateway assigns the transmission channels to its governed nodes. We propose that spectrum management algorithms shall be hosted by the gateway, as it can have a constant power source (e.g., mains). On the other hand, the nodes, which are mainly battery operated, can have only limited functionality related to sensing the data and transmitting it to the gateway. This saves the node’s battery to a great deal and is very desirable for many applications of sensor networks.

We have previously proposed a dynamic channel allocation algorithm for cognitive radio networks [31]. To turn the WBAN into a cognitive body area network (C-BAN), we have revised the algorithm and made it more dynamic and robust for the resource limited computational platform.

#### 3.5.1. Reinforcement Learning—Channel Assignment Algorithm (RL-CAA)

The RL-CAA is based on reinforcement learning, which itself comes from the field of artificial intelligence and machine learning. The RL-CAA builds upon a Bernoulli distribution and its internal architecture is based on the Bernoulli random variables and probabilities. This internal part of the algorithm is called the Bernoulli logic unit [32,33]. The algorithm works following an unsupervised learning approach and interacts with the target environment and considers the feedback. The algorithm interaction with the environment is based on feedback and M input signals. An input signal x is biased by the weighting factor w, which are real vectors containing input variables for the algorithm and their corresponding weights. The inputs and their weights are combined as per Equation (2) in a scalar quantity z:

(2) $$z=\overset{M}{\underset{i=1}{\sum }}w_{i}x_{i}$$

where M is total number of channels; z is then subjected to a Bernoulli logistic function given in Equation (3):

(3) $$p=f\left(z\right)=\frac{1}{1+e^{-z}}$$

Then the algorithm updates its internal probabilities and context variables (i.e., x and w) based on that feedback. A probability bias σ is added to p so that it may not converge to 0. Based on the feedback signal, a decision is made according to the context in which RL algorithm is being used. The feedback is termed reward signal r(t) in the RL terminology. The instantaneous reward signal in our simulation domain is given in Equation (4):

(4) $$r\left(t\right)=\{\begin{array}{c} 0,\;if\;Throughput<TH\;or\;SNR<0\;or\;interference>I_{\mathrm{Th}}\\ \bar{\mathrm{SNR}},\;otherwise \end{array}$$

where the throughput threshold, i.e., TH, depends upon the total number of nodes at the gateway (Table 4) and $I_{\mathrm{Th}}$ is the interference threshold. The reward value is the average signal to noise ratio (SNR) of the channel. The output of the algorithm is a Bernoulli random variable (Equation (5)). At the start of the simulations, each gateway executes the RL-CAA in order to find the optimal channel allocation for the data transmission. The RL-CAA traverses all the available channels; the channel(s) which meet the criteria given in Equation (5) are then selected:

(5) $$y=\{\begin{array}{c} 0,\;if\;r\left(t\right)\leq 0\\ 1,\;if\;r\left(t\right)>0 \end{array}$$

The probability mass function representing the probability p of output y is described in Equation (6):

(6) $$g\left(y,p\right)=\{\begin{array}{c} 1-p,\;if\;y=0\\ p,\;if\;y=1 \end{array}$$

To simplify, y is a two action learning outcome of the RL process. The learning of the agent can be condensed in the weighting vector so that at each time step t, the agent learns by updating its weighting vector using Equation (7) and any instantaneous change in the individual weight is calculated by Equation (8):

(7) w(t)=w(t−1)+Δw(t)

(8) $$\Delta w_{i}\left(t\right)=\alpha \left(t\right)\left[r\left(t\right)-\bar{r}\left(t-1\right)\right]\left[y\left(t-1\right)-p\left(t-1\right)\right]x_{i}\left(t-1\right)$$

where α(t) > 0 is called the learning rate, r(t) is the reward returned by the environment at any instant (step) of time t, and $\bar{r}\left(t\right)$ is the average reward which is obtained as shown in Equation (9):

(9) $$\bar{r}\left(t\right)=\beta r\left(t\right)+\left(1-\beta \right)\bar{r}\left(t-1\right)$$

where 0<β≤1 is called the reward memory factor of the RL. Low values of β assures enough memory of the past rewards. Decreasing the learning rate α with the RL steps improves the convergences speed of the algorithm. Thus, the learning rate is linearly decreased as given in Equation (10):

(10) α(t)=α(t−1)−Δ

where Δ should be small enough to assure a smooth transition between steps and negative values for α should be avoided.

If any channel is giving a throughput below the target throughput, or if the channel is experiencing co-channel interference, the reward function returns zero (Equation (4)) and the RL-CAA discards that channel and continues its exploration. Once the algorithm has converged, the set of optimal channels is known (Equation (11)): duplicated number

(11) $$Y=\left\{y_{1},y_{2},\ldots y_{M}\right\}$$

where $y_{i}$ represents the outcome of the RL-CAA for an individual channel. These channels are subsequently assigned to the sensor nodes and the gateway starts receiving data from the nodes. At any instant, due to the change in throughput requirements (either the target throughput has been changed or the traffic load has changed in the zone due to handovers) the particular gateway executes the RL-CAA to look for the new optimal channel allocations to satisfy its governed nodes. Some of the key parameters of the RL-CAA are presented in the Table 3. The key steps of the RL-CAA are listed in Algorithm 1 (note that this is a revised version of [34] with more focus on QoS requirements and co-channel interference).

**Algorithm 1:** The key steps of the RL-CAA.1. **REPEAT UNTIL** RL Convergence **OR** RL Evaluation steps 2. Sense the available channels’ conditions, i.e., SNR, interference 3. Calculate instantaneous reward, r(t), as per Equation (4) 4. Update average reward, $\bar{r}\left(t\right)$, as per Equation (9) 5. **FOR** i = 1 to M 6.  Update: x, w, p 7. **END FOR**
8. **FOR** i = 1 to M 9.  **IF** p_i_ > 0.5 10.   y_i_ = 1, i.e., assign the channel 11.  **ELSE**
12.   y_i_ = 0, i.e., do not assign the channel 13.  **END IF** 14. **END FOR** 15. **END REPEAT** 16. **FOR** i = 1 to M 17. C_i_ = Channel_Capacity(y_i_) 18. **IF** TotalTH < Req_TH 19.  TotalTH = TotalTH + C_i_ 20. **ELSE** 21.  y_i_ = 0 22. **END IF**
23. **END FOR**

#### 3.5.2. Static Channel Assignment (SCA)

In order to evaluate (later in Section 4) the performance of the proposed RL-CAA, we compare it to the SCA even selection algorithm [35]. The algorithm has been modified in order to comply with our application domain. The operation of the algorithm is as follows. The gateway monitors the available channels. The channels are assigned in an increasing order based on the gateway ID, irrespective of the channel conditions, e.g., SNR, interference, etc. For example, if Gateway 1 needs to allocate a channel and if Channel 1 is currently assigned, then Channel 2 will be assigned to Gateway 1 and Gateway 2 will take Channel 3 and so on.

### 3.6. Transmission Power

WBANs are distributed networks and often a large number of networks have to co-exist close to each other. Each network has its own transmission power and it is practically very hard to maintain cooperation among all the WBANs to avoid co-channel interferences. Traditionally assigned transmission powers are not very optimized, so there is a big room for power optimization in WBANs. These traditional transmission power algorithms can generate tremendous amounts of interferences in the neighboring WBANs [21,36]. We propose a novel power allocation algorithm to reduce the interference among gateways (which are acting as base stations for all control operations for the individual WBANs). Each gateway is governing various WBANs in its vicinity. The primary convergence criteria for the power optimization is interference minimization.

The transmission power is calculated based on the desired interference level at the boundary of the WBAN, i.e., edge of a zone. The algorithm is derived from the convex optimization and is based on the illumination problem [37]. To calculate the transmission power, the interference boundary, i.e., perimeter of the zone, is divided into equally spaced linear small k patches. The interference received at any patch should not be greater than I_des_, which is known as the interference threshold to avoid any interference to the neighboring WBANs. Equation (12) states the received power/interference level, I_k_, at the center of each patch of interference boundary:

(12) $$I_{k}=\sum_{j=1}^{m}a_{\mathrm{kj}}P_{T},$$

where m is the number of transmitters (base stations). Since there is only one gateway serving the zone, here m = 1 and P_T_ is the optimal transmission power of the transmitter. a_kj_ is the propagation losses between transmitter and the receiving path k, where the interference level is calculated. a_kj_ is calculated from the path loss and channel model presented in Section 3.3. Since the interference threshold, I_des_, is known (it is determined empirically), the algorithm calculates the transmission power meeting this interference criterion for the given gateway. For optimization purposes, the objective function is formulated as shown in Equation (13):

Minimize:

(13) $$\max_{k=1,\ldots ,n}\left|\log I_{k}-\log I_{\mathrm{des}}\right|,$$

Subject to:

(14) $$0\leq p_{j}\leq p_{\max},$$

## 4. Simulation Results

In this section, simulation results for our proposed algorithms are presented. At the beginning of the simulation, the WBANs are deployed, i.e., the gateway and the nodes are placed in an area of 10 × 10 m^2^. Each zone of 5 × 5 m^2^ starts with an equal number of nodes. In the cases when mobility is also considered, the nodes can move across all the zones freely and randomly. The key simulation parameters are listed in Table 4.

We have performed the simulations for various scenarios modeling realistic nursing home activity patterns. These scenarios are modeled from the idlest time (when all nodes are stationary) to the busiest activity time, i.e., all nodes are mobile. These scenarios are:

- Case A: All the nodes are stationary. This case depicts that all patients are sleeping at night. They are not moving so there is no traffic change during this time.
- Case B: This corresponds to 100% mobility. All the nodes are moving. This situation corresponds to the busy working hours when all patients have to perform routine tasks.
- Case C: In this case, eight nodes are moving and four nodes are stationary.
- Case D: In this case, four nodes are moving and eight nodes are stationary.

Cases C and D correspond to the situations when some patients are resting and some of them are moving around, for example during the first hours of the working day, after lunch, or evening before bedtime. To evaluate the performance of our proposed RL-CAA, we compare its results with the classical static channel assignment scheme SCA. The RL-CAA is also analyzed based on various QoS requirements. The analysis of the simulation results are done on the basis of three performance parameters; (i) bit error rate (BER), which is the average bits in error received at the gateway; (ii) throughput, average throughput in bits per second (bps) available at the gateway; and (iii) dissatisfaction probability. The data involved in the WBANs can be quite critical depending upon the nature of the application, so the users (nodes) must have sufficient quality of service (QoS) to transmit their data properly. Dissatisfaction can give an indication of the QoS at any given time. Here, the dissatisfaction probability is defined as the percentage of time in which the users’ QoS (i.e., user throughput) is below the target (user) throughput. This target throughput is often referred as satisfaction throughput. Here the satisfaction throughput is chosen as 971.4 kbps, i.e., the maximum allowed by the IEEE 802.15.6 standard.

### 4.1. Case A

**Figure 5:**
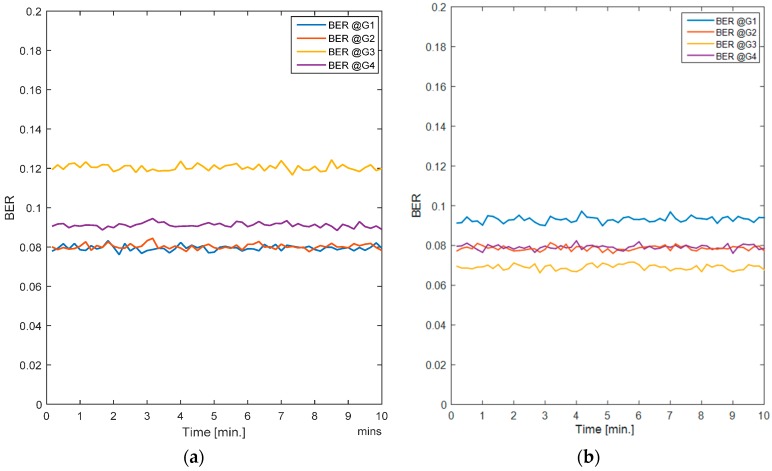
(**a**) Average BER for the SCA algorithm; (**b**) Average BER for the RL-CAA algorithm.

**Figure 6:**
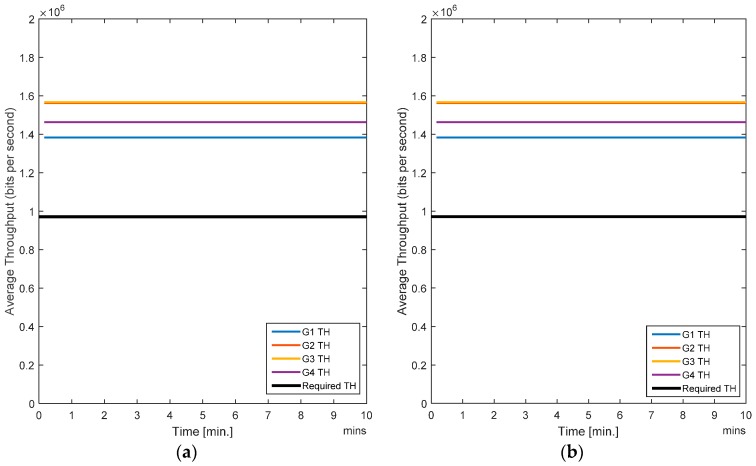
(**a**) Average throughput for the SCA algorithm; (**b**) Average throughput for the RL-CAA algorithm.

In this case, all nodes are stationary. Figure 5a shows the averaged BER per gateway for the SCA and Figure 5b shows the average BER for the RL-CAA. The average error rate for SCA is a little over 10%, whereas it is less than 10% for each gateway with the RL-CAA. The average throughput per gateway provided by both algorithms is quite similar, as shown in Figure 6a,b.

All the users have acceptable QoS so no dissatisfaction is observed (probability = 0). The SCA is a static algorithm whereas the RL-CAA is a dynamic and robust algorithm (i.e., it can promptly act on any change in the network parameters and can adjust accordingly to provide sufficient QoS), and it is suited for scenarios with changing traffic requirements. In case of a static scenario, there are no much benefits with the RL-CAA.

### 4.2. Case B

**Figure 7:**
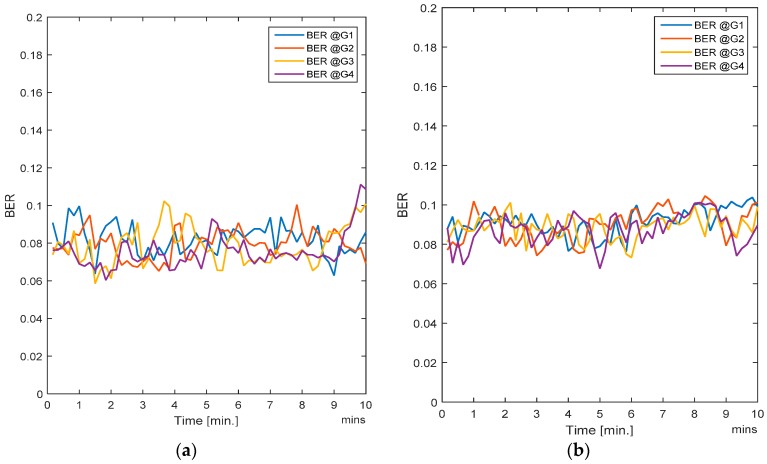
(**a**) Average BER for the SCA algorithm; (**b**) Average BER for the RL-CAA algorithm.

In this case, all the users (i.e., nodes) are moving. Since handovers are allowed from one zone to another, the users move freely and randomly across the entire region. Traffic load is also changing randomly as the nodes are entering or leaving a zone. The RL-CAA is a dynamic and robust algorithm which can confront the traffic change instantly, but it has a downside. More dynamics mean that the RL-CAA will undergo exploration of new channels as soon as the throughput goes below the throughput threshold. The RL-CAA is computationally complex and it has a learning curve before it can converge to new channel assignments. Hence, there is a tradeoff between the impact on the desired QoS and the RL-CAA execution overheads. We do not require too frequent execution of the RL-CAA, so we set a 5% margin in throughput requirements. As the gateway throughput falls 5% below the target throughput, the RL-CAA is executed by the gateway and the new channels are then assigned. Figure 7a,b show the average BER of the SCA and the RL-CAA, respectively.

**Figure 8:**
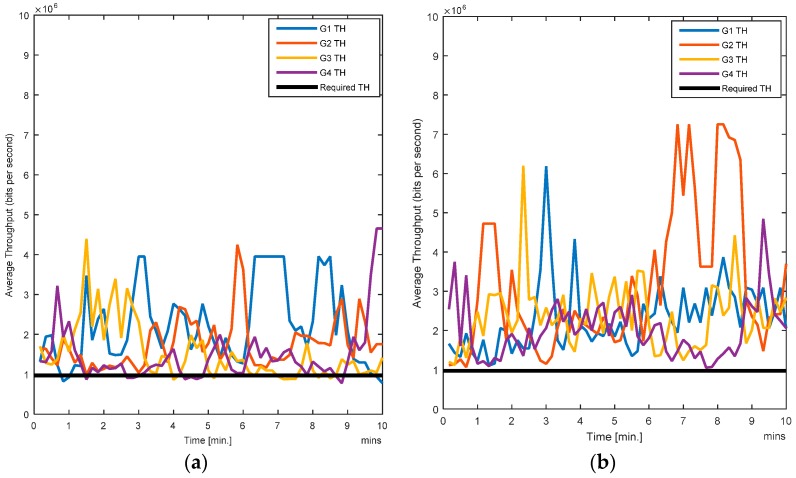
(**a**) Average throughput for the SCA algorithm; (**b**) Average throughput for the RL-CAA algorithm.

**Figure 9:**
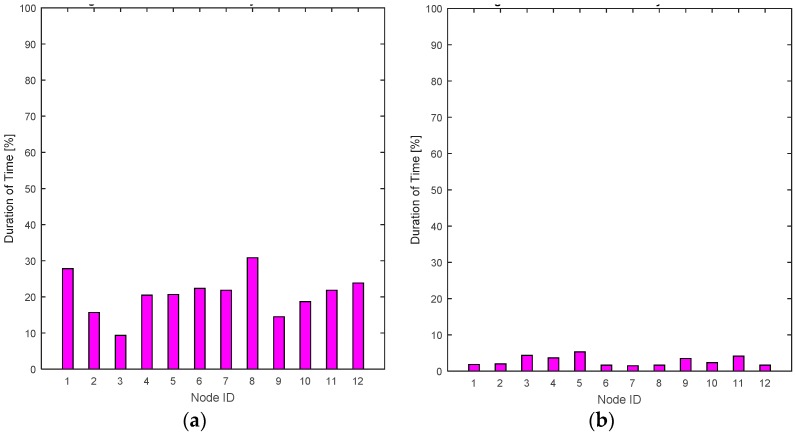

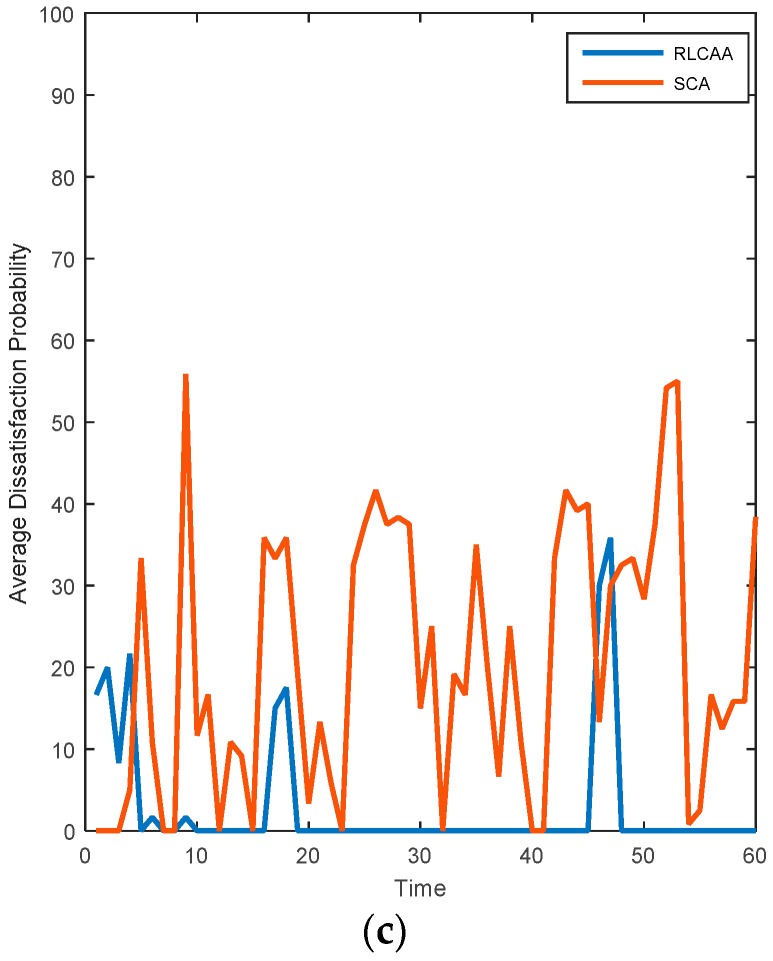
(**a**) Individual nodes dissatisfaction probabilities for the SCA algorithm; (**b**) Individual nodes dissatisfaction probabilities for the RL-CAA algorithm; (**c**) Comparison of the RL-CAA and SCA based total dissatisfaction probabilities averaged over time.

The RL-CAA is giving much consistent BER under 10% while SCA is fluctuating around 10%. The average throughput available at each gate for the SCA and the RL-CAA are shown in Figure 8a,b, respectively. The RL-CAA provides better distributed and higher throughput performance (reaches over 7 Mbps instantaneous throughput). The dissatisfaction probabilities, as experienced by the individual nodes, are given in Figure 9a,b for the SCA and RL-CAA, respectively.

The RL-CAA is giving much consistent BER under 10% while SCA is fluctuating around 10%. The average throughput available at each gate for the SCA and the RL-CAA are shown in Figure 8a,b, respectively. The RL-CAA provides better distributed and higher throughput performance (reaches over 7 Mbps instantaneous throughput).

The dissatisfaction probabilities, as experienced by the individual nodes, are given in Figure 9a,b for the SCA and RL-CAA, respectively.The RL-CAA provides better QoS satisfaction as compared to the SCA. This can also be seen from Figure 9c, where the total dissatisfaction probabilities averaged over time for all nodes is presented for both algorithms. The RL-CAA provides, on average, a QoS satisfaction to individual nodes 97.2% of the time, while the SCA can only provide, on average, QoS satisfaction 79% of the time. These results consolidate the hypothesis that the proposed RL-CAA is highly effective in traffic changing environment and can accommodate handovers.

### 4.3. Case C

**Figure 10:**
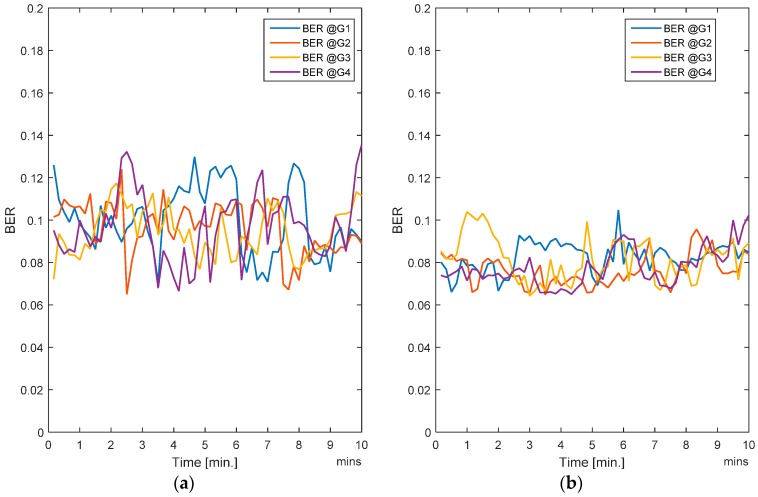
(**a**) Average BER for the SCA algorithm; (**b**) Average BER for the RL-CAA algorithm.

In this scenario, eight nodes are moving while four are stationary. The average BER, as observed at each gateway, is shown in Figure 10a,b for SCA and RL-CAA, respectively.

**Figure 11:**
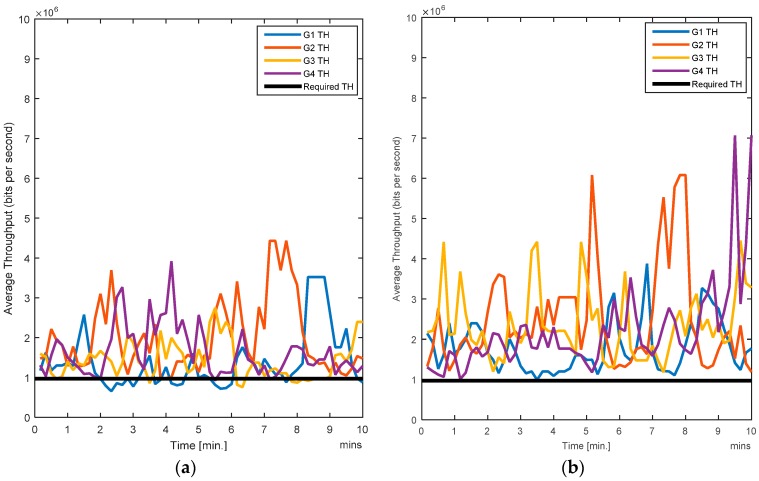
(**a**) Average throughput for the SCA algorithm; (**b**) Average throughput for the RL-CAA algorithm.

**Figure 12:**
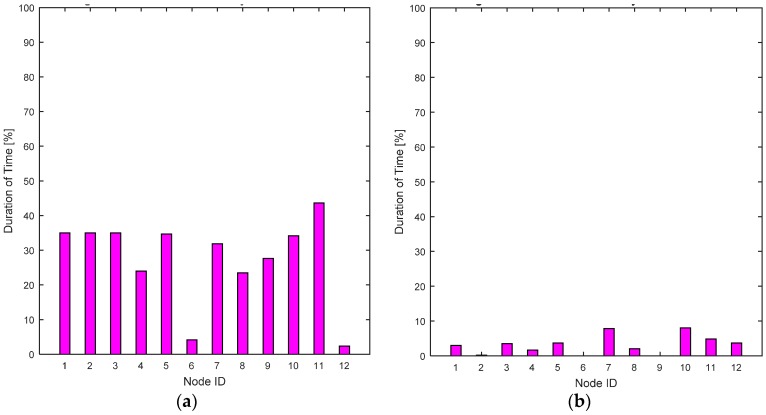

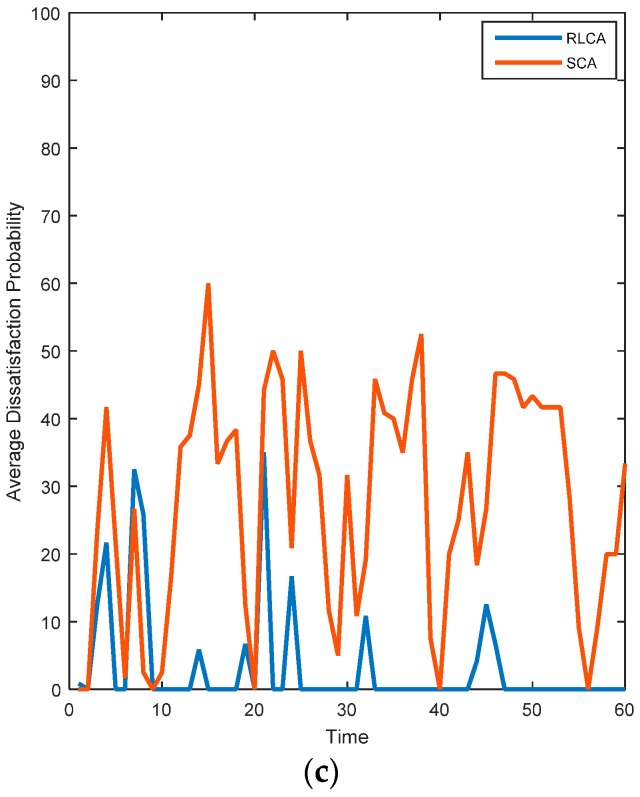
(**a**) Individual nodes dissatisfaction probabilities for the SCA algorithm; (**b**) Individual nodes dissatisfaction probabilities for the RL-CAA algorithm; (**c**) Comparison of the RL-CAA and SCA based total dissatisfaction probabilities averaged over time.

The RL-CAA is giving a below 10% error rate on average and a few fluctuations giving an error rate over 10%. The SCA’s BER is over 10% for all gateways. The RL-CAA is providing better throughput per gateway as compared to SCA. For a short period, G4 experiences a throughput over 7 Mbps (Figure 11a,b). From Figure 12a,b, it is clear that RL-CAA provides better QoS satisfaction and little dissatisfaction is observed. From Figure 12c, it can be observed that there are short periods when the total system faces dissatisfaction. The RL-CAA in general provides better user QoS satisfaction.

### 4.4. Case D

**Figure 13:**
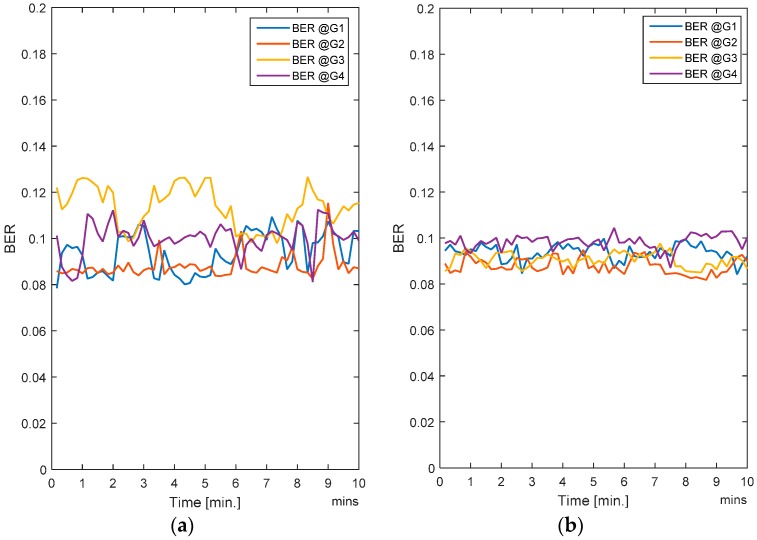
(**a**) Average BER for the SCA algorithm; (**b**) Average BER for the RL-CAA algorithm.

**Figure 14:**
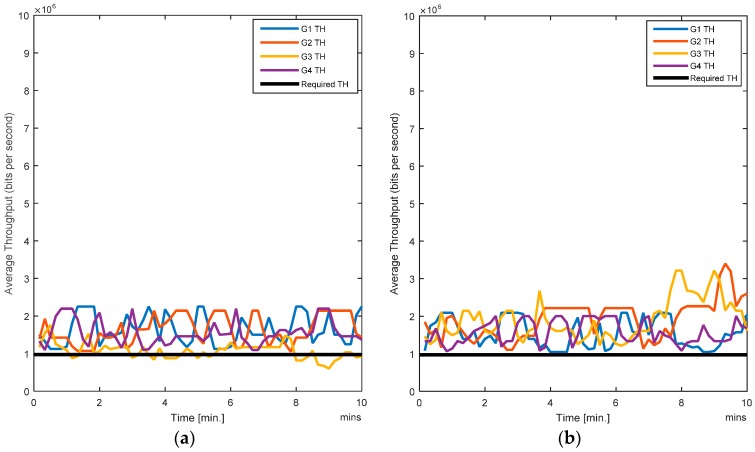
(**a**) Average throughput for the SCA algorithm; (**b**) Average throughput for the RL-CAA algorithm.

In this scenario, four users are mobile while eight users are stationary. The average BER values, shown in Figure 13a,b, illustrate that the RL-CAA is consistent in providing a ~10% error rate while SCA is giving slightly more than 10%. Since few nodes are mobile, less handovers are occurring, therefore throughputs observed at each gateway are more consistent, as shown in Figure 14a,b.

**Figure 15:**
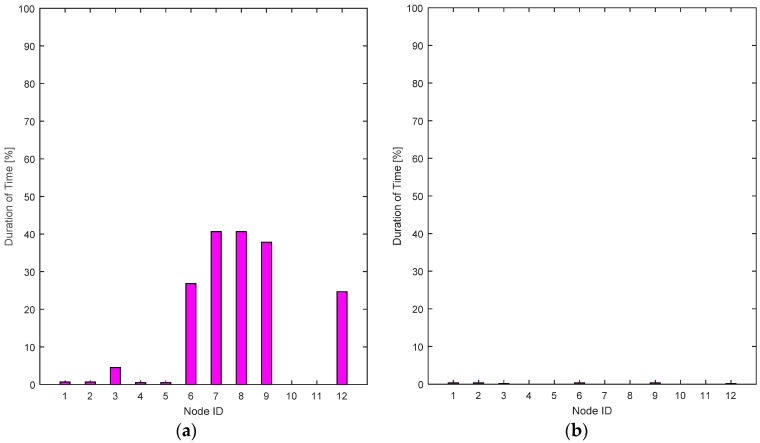

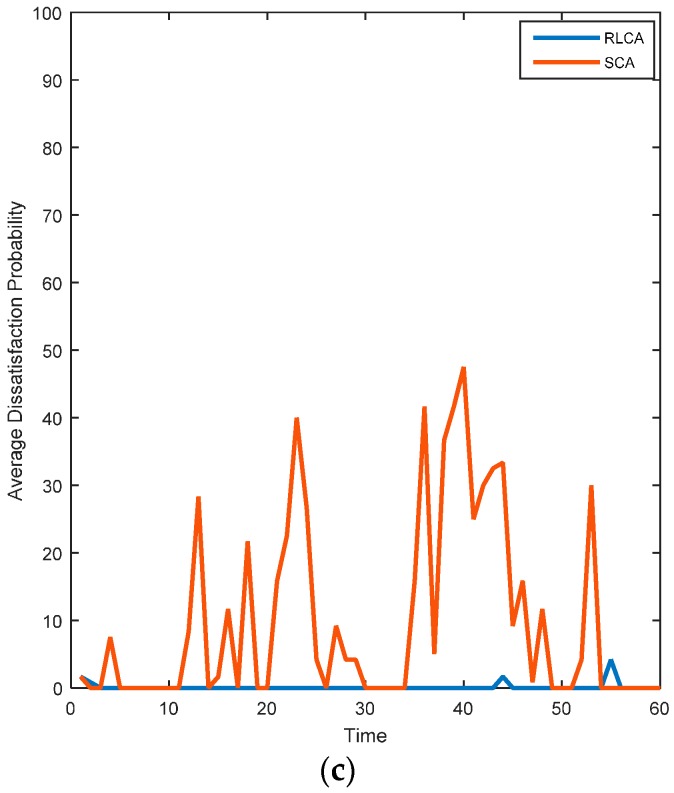
(**a**) Individual nodes dissatisfaction probabilities for the SCA algorithm; (**b**) Individual nodes dissatisfaction probabilities for the RL-CAA algorithm; (**c**) Comparison of the RL-CAA and SCA based total dissatisfaction probabilities averaged over time.

The RL-CAA provides a better overall throughput, e.g., at G2 and G3 the average throughput is over 3 Mbps. The individual dissatisfaction probability for the RL-CAA is almost zero (Figure 15b). The SCA provides poor QoS and the nodes are dissatisfied. The total average system dissatisfaction probabilities of the RL-CAA and the SCA are compared in Figure 15c. The overall dissatisfaction for the RL-CAA is negligible.

As previously discussed, the RL-CAA is a computationally expensive algorithm. We managed to minimize its execution time in a traffic-changing environment by introducing a throughput margin for the acceptable throughput. The lower the throughput margin, the higher the number of executions of the RL-CAA in a given amount of time. There is a tradeoff between QoS requirements and the acceptable throughput margin. The results presented above correspond to a 5% margin for the throughput.

Based on these simulated results, the proposed RL-CAA gives an overall better performance. In terms of BER, the RL-CAA performs slightly better than the SCA; we can summarize that the RL-CAA and the SCA exhibits similar behaviors. However, the RL-CAA gives better performance in terms of throughput and dissatisfaction probabilities, which is the main aim in this work.

**Figure 16:**
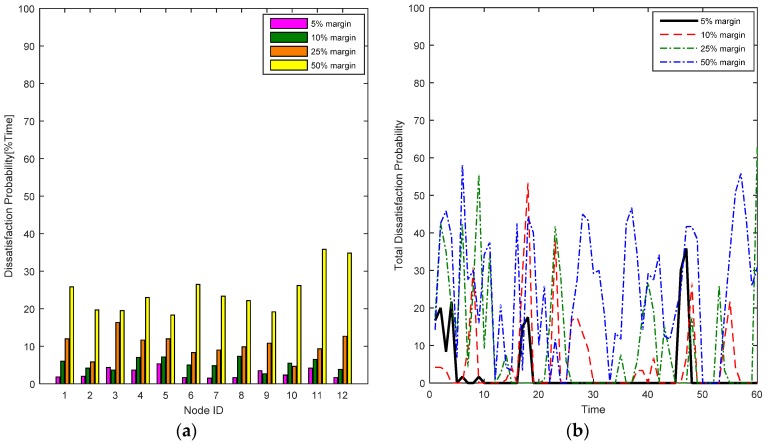
(**a**) Comparison of individual dissatisfaction probabilities of 5–50% throughput margin values; (**b**) Comparison of total averaged dissatisfaction probabilities for 5–50% throughput margin values.

We analyze the results of the RL-CAA with different throughput margins. Case B is the most dynamic with the highest mobility, handovers and changing traffic, hence it is the most challenging case for the algorithm in terms of complexity, dynamism and robustness. The RL-CAA is analyzed only for the QoS satisfaction for the case B, for 5%, 10%, 25% and 50% throughput margin. It is evident from Figure 16a that the 5% margin gives the best individual nodes’ QoS satisfaction, i.e., the individual dissatisfaction probability is lowest.

This aspect can also be presented in the form of the total averaged dissatisfaction probability of all the margin values, as plotted in Figure 16b. The 5% margin shows the lowest dissatisfaction probability among all. The 50% throughput margin yields almost the same dissatisfaction probability as the SCA (refer to Figure 8 and Figure 9). The results show that for the presented experiments, the RL-CAA yields the best results for channel allocation in a highly dynamic environment and that a 5% margin is good tradeoff to achieve the benefits of unsupervised learning and making the WBAN as C-BAN.

To evaluate our power optimization algorithm, we take the example of Zone 1 with gateway G1. We use three reference users located at A, B and C (Figure 2). These users are located at the edges of their zones, which is also the edge of Zone 1. This is the closest point where these users can come into the coverage radius of G1. The available transmission power ranges from −13 to 3 dB (Table 1). In a conventional power assignment scheme, the maximum available power (3 dBm) is assigned to the base station, i.e., the gateway. For simplicity, let’s only take the case of direct LOS; the users at location A, B and C are receiving interference of −46.7688 dBm, −49.5413 dBm and −46.7688 dBm, respectively. With our power optimization algorithm, the same users receive −51.266 dBm at location A, −54.0386 dBm at location B, and −51.266 dBm at location C, when they are in direct LOS with G1. These results illustrate that our power optimization algorithm improve the performance when co-channel interferences exist and the users receive much less interference from neighboring zones. Our proposed algorithm sets the transmission power to −1.4973 dBm for G1. This gives 4.479 dBm lower interference at these reference points.

## 5. Conclusions

In this paper, a cognitive and dynamic channel algorithm named RL-CAA has been presented. An optimized channel can give better throughput and QoS as shown by our simulation results. In static environments, where the sensor nodes are not much mobile, computationally expensive algorithms are not much required as can be seen in our simulation results for Case A. In highly demanding environments, which can be due to the change of the throughput requirements on the run or due to the sensor nodes’ frequent handovers, the RL-CAA is a better choice for C-BANs. The static channel assignment algorithm cannot keep up with the requirements of volatile network conditions. However, the RL-CAA has high computational overheads, i.e., complexity, memory requirements, execution time, etc. Hence, it is recommended to use it in an application where the throughput and QoS requirements must not be compromised.

Our proposed RL-CAA presented here is evaluated for a specific health related application, but it is largely application-independent. It can be used in other types of sensor networks applications with little or no modification to the internal structure of the algorithm. Due to its unsupervised learning approach, the RL-CAA is well suited for distributed WSNs where there is no need for a central controller or coordinator to supervise spectrum sharing.

The power optimization algorithm gives better performance by reducing the transmission power and better performance in terms of interference. The radio module is one key component which drains the batteries of the sensor nodes. Our proposed power optimization algorithm saves up to 4.4973 dBm in transmission power. Reducing the transmission power can increase the battery life of the sensor nodes. The algorithm is most suited for base stations or gateway nodes, which are not mobile or not changing their positions over longer periods. It is not computationally intensive so it is quite suitable for resource-limited platforms. The downside of the algorithm is that it requires the path loss matrix to be calculated and stored in memory before executing the power optimization algorithm.

## 6. Future Work

The work presented in this paper can be seen as a starting point towards the research and development of C-BANs. This work can be extended toward more diverse scenarios and applications of WBANs. Considering the pace of development in modern technologies, it is expected that the size of the sensors will reduce dramatically. Hence, it will be possible in the future to carry or wear many more sensor nodes on the body. Such dense deployment of the sensor nodes in a WBAN will create new challenges in terms of spectrum management, e.g., interference, QoS, etc. Traditional approaches will no longer be very effective to encounter such challenges. The cognitive capabilities in the C-BANs will be suitable candidate to combat these challenges. Since most of the WBANs are proposed or designed for the ISM band and with growing diverse services in this unlicensed band, more cognitive functionalities will be required at various layers in the C-BANs.

Interference is a major hindrance for the QoS of the WBANs. With the introduction of C-BANs, efficient and opportunistic spectrum management algorithms can be proposed. In this work, inter-gateway interference avoidance has been proposed through power control. In the future, inter-WBAN and beyond-WBAN interferences could be avoided with cognitive capabilities of future C-BANs.

Channel modeling has been a tough subject for WBANs’ researchers. Off-body and body-to-body channel modeling has been very little investigated in the literature. In this work, an off-body mathematical channel model has been proposed based on the body postures and shadowing resulting from various factors. A more comprehensive model of the off-body channel link based on the antenna design and position could be a future investigation task.

Finally, the algorithm presented in this work (i.e., the RL-CAA) has been proposed for channel allocation and simulation results show it as a promising candidate for future C-BANs. However, hardware implementation and design considerations for resource constrained platforms (in particular their computational power and energy conservation issues) remain to be investigated; for this, further modifications and optimizations to the proposed approach would be needed to improve the matching between the algorithms and architecture.

## Figures and Tables

**Table 1:** System features and requirements.

Features	Values
Frequency band	2.4–2.483 GHz
Number of channels	79 (according to ISM band)
Payload	0–2040 bits
Channel bandwidth	1 MHz
Data rates	121.4 Kbps–971.4 Kbps
Modulation Scheme	DBPSK/DQPSK

**Table 2:** Channel model values of the polynomial constants.

Constants	LOS	NLOS
a	2.45	3.673
b	−17.99	−30.61
c	43.95	76.94
d	16.05	9.48

**Table 3:** RL-CAA simulations parameters.

RL-CAA Parameters	Values
RL Evaluation Steps	500
RL Convergence Steps	50
α (RL learning rate)	100
β (reward memory factor)	0.01
σ (probability bias)	0.05

**Table 4:** Simulation parameters.

Simulation Parameters	Values
Frame size	2040 bits
Transmission power (fixed power case)	3 dBm
Node Throughput	971.4 Kbps
Total Nodes per Gateway	3
Zone Area	25 m^2^
Total Region	100 m^2^
Maximum distance between a node—gateway (before handover may occur)	3.54 m
Total Simulation Time	600 s
Simulation statistics averaging period	10 s
